# LRP-1: A Checkpoint for the Extracellular Matrix Proteolysis

**DOI:** 10.1155/2013/152163

**Published:** 2013-07-10

**Authors:** Nicolas Etique, Laurie Verzeaux, Stéphane Dedieu, Hervé Emonard

**Affiliations:** CNRS FRE 3481 MEDyC (Matrice Extracellulaire et Dynamique Cellulaire), Laboratoire SiRMa (Signalisation et Récepteurs Matriciels), Université de Reims Champagne-Ardenne (URCA), Moulin de la Housse, Bât. 18, Chemin des Rouliers, BP 1039, 51687 Reims Cedex 2, France

## Abstract

Low-density lipoprotein receptor-related protein-(LRP-1) is a large endocytic receptor that binds more than 35 ligands and exhibits signaling properties. Proteinases capable of degrading extracellular matrix (ECM), called matrix proteinases in this paper, are mainly serine proteinases: the activators of plasminogen into plasmin, tissue-type (tPA) and urokinase-type (uPA) plasminogen activators, and the members of the matrix metalloproteinase (MMP) family. LRP-1 is responsible for clearing matrix proteinases, complexed or not with inhibitors. This paper attempts to summarize some aspects on the cellular and molecular bases of endocytic and signaling functions of LRP-1 that modulate extra- and pericellular levels of matrix proteinases.

## 1. Introduction

Extracellular matrix (ECM) remodeling occurs in both physiological and pathological situations [1]. Tissue homeostasis depends on a strict equilibrium between synthesis and degradation of ECM macromolecules. In contrast, fibrotic pathologies are classically related to a defect or an increased ECM breakdown, while an excessive proteolytic degradation is the hallmark of inflammatory processes or tumor invasion. Numerous proteolytic enzymes are able to degrade ECM macromolecules, including the serine proteinases tissue-type plasminogen activator (tPA) and urokinase-type plasminogen activator (uPA) [2] and the members of the matrix metalloproteinase (MMP) family [3]. 

A series of specific or nonspecific inhibitors controls the activities of these powerful catalytic enzymes. Thus, the pan-protease inhibitor *α*2-macroglobulin (*α*2M) binds to and inhibits active members of the four classes of proteolytic enzymes [4]. More specifically, the serine proteinase inhibitors (serpins) and the plasminogen activator inhibitors (PAI) 1 and 2 block the activity of tPA and uPA [5]. Tissue inhibitors of metalloproteinases (TIMPs) inhibit the activity of MMPs [6] and also of adamalysins (a disintegrin and metalloproteinases, ADAMs) [7]. Besides this level of control, receptor-mediated endocytosis is an emergent and efficient biological mechanism to regulate extra- or pericellular levels of proteolytic enzymes by internalizing them for catabolism in lysosomes [8]. This paper briefly describes the main molecules involved in these events and reviews the different roles of low-density lipoprotein (LDL) receptor-related protein-(LRP-1) in controlling extracellular matrix remodeling.

## 2. Plasminogen Activators and Their Inhibitors

Urokinase-type plasminogen activator (uPA) and tissue-type PA (tPA) are serine proteinases that catalyze the conversion of the zymogen plasminogen to the active serine proteinase plasmin [9]. Plasmin degrades numerous ECM macromolecules including laminin, fibronectin, and proteoglycans, triggers the activation of pro-MMPs, and activates or releases growth factors from ECM including latent-transforming growth factor *β* and vascular endothelial growth factor. Both *α*2M and the serpin *α*2-antiplasmin inhibit its activities [10]. Pro-uPA is synthesized as a one-chain molecule that is cleaved at a single peptide bond (K158-I159 in human uPA) by various proteases including plasmin to give active two-chain uPA of 55 kDa. Human tPA was first purified as a single-chain form of approximately 70 kDa. A limited attack of the R275-I276 bond by plasmin generates a two-chain tPA. The plasminogen activation activity of single-chain tPA is 10- to 50-fold lower than that of the two-chain form [2]. The PA inhibitors PAI-1 and PAI-2 efficiently inhibited tPA and uPA catalytic activities [11]. 

 The binding of uPA to its cell-surface receptor (uPAR) increases the affinity of uPAR for vitronectin and integrins, thus promoting cell adhesion, [12]. Interestingly enough by disrupting these interactions, PAI-1 detaches cells not only from vitronectin but also from fibronectin and collagen matrices [13]. This deadhesive property exhibited by PAI-1 could explain, at least partly, why paradoxically PAI-1 appears to be essential for cancer cell invasion and angiogenesis [14]. 

## 3. Matrix Metalloproteinases and Their Inhibitors

MMPs are the major matrix-degrading proteases due to the wide variety of their substrates and their role in numerous physiopathological processes [15, 16]. They belong to a large family of zinc-dependent endopeptidases. In humans, MMPs are represented by 23 members divided into two groups based on their localization (secreted or membrane-bound) or in five groups based on their domain organization and their substrate preference (collagenases, gelatinases, stromelysins, matrilysins, and membrane-type) [3, 17]. The general structure of MMPs consists in three domains that are common to almost all MMPs: the prodomain of about 80 amino acids, the catalytic metalloproteinase domain of about 170 amino acids, and the hemopexin domain of about 200 amino acids (except for MMP-7, -26, and -23). MMPs are secreted as a proenzyme, an enzymatically inactive state that results from the interaction between the “cysteine switch” motif in the prodomain and the zinc ion of the catalytic site [18]. The activation of these zymogens is an important regulatory step of MMP activity and occurs after the disruption of this interaction [15]. This process requires the proteolytic removal of the pro-domain by intracellular convertases such as furin or by extracellular proteinases (MMPs, plasmin,…, etc.). A chemical perturbation of the cysteine-zinc interaction by SH reagents, by chaotropic agents (*in vitro*), or by antioxidant has been shown as sufficient to activate proMMPs [18]. 

After their activation, MMPs are regulated by two major types of endogenous inhibitors: *α*2M and TIMPs [18]. *α*2M is a plasma glycoprotein produced in the liver. Four nearly identical, disulfide-bonded domains of 180 kDa compose this 772 kDa protein. Inhibition mechanism involves the presentation of a cleavable region that, once proteolytically cleaved, induces a conformational change entrapping the proteinase that becomes covalently anchored by transacylation. Such a molecular complex is rapidly cleared by LRP-1-mediated endocytosis [19].

TIMPs are 184–194 amino acid proteins that have been described to form 1 : 1 stoichiometric complexes with active MMPs leading to the inhibition of their proteolytic activity. Four TIMPs (TIMP-1, -2, -3, and -4) have been identified in humans, inhibiting all MMPs tested so far, except TIMP-1 that was reported as being a poor inhibitor for MT1-MMP, MT3-MMP, MT5-MMP, and MMP-19 [6, 18, 19]. All structurally characterized inhibitory TIMP-metalloproteinase complexes are closely similar. Within the metalloproteinase active site, the catalytic zinc atom is chelated by the N-terminal amino group and the carbonyl group of cysteine 1 [20]. TIMPs are also able to interact with proMMPs: TIMP-2, TIMP-3 or TIMP-4 with proMMP-2 and TIMP-1 or TIMP-3, with proMMP-9 [20]. These complexes are stabilized by interaction between the TIMP C-terminal domain and hemopexin domain of the zymogen. Since these interactions do not involve the N-terminal domain of the TIMP, such molecular complexes are capable of interacting with a second MMP molecule. Except for the role of proMMP-2-TIMP-2 in the MT1-MMP-mediated activation of proMMP-2 [21], their functional significance remains unclear [20].

More recently, TIMPs have been reported to induce various biological processes (cell survival, differentiation, epithelial-mesenchymal transition,…, etc.) independently from their MMP-inhibitory activity [22, 23]. These effects involved an interaction with specific cell-surface receptors leading to signaling pathway activation. For example, TIMP-1 promotes cell survival in erythroleukemic cells after binding with a CD44/proMMP-9 complex receptor [24, 25] and in breast and lung epithelial cells after interacting with a CD63/integrin-*β*1 complex receptor [26, 27].

## 4. Low-Density Lipoprotein Receptor-Related Protein-1

### 4.1. General Features

 LRP-1 is the first member of a receptor family related to the LDL receptor [28]. The receptor for *α*2M-proteinase complexes [29] and CD91, which interacts with heat-shock proteins at the surface of antigen-presenting cells [30], corresponds to LRP-1. It is synthesized as a single-chain molecule processed by furin in the trans-Golgi compartment into a 515 kDa *α*-chain and an 85 kDa *β*-chain which remain non-covalently associated at the cell surface [8]. The extracellular *α*-chain contains four basic amino acid residue-rich domains that interact with a number of ligands including proteins involved in lipoprotein metabolism, ECM proteins, growth factors, proteinases, and proteinase-inhibitor complexes. The transmembrane *β*-chain contains a cytoplasmic tail of 100 amino-acid residues including two NPxY motifs, necessary to trigger endocytosis and capable of interacting with many adaptors and signaling proteins. 

 The endocytic clearance of various ligands and signaling properties confer a main role to LRP-1 in a variety of pathophysiological processes including lipid metabolism, neurodegenerative diseases, blood-brain-barrier integrity, atherosclerosis, and cancer [8]. The importance of LRP-1 is confirmed by the lethality of mice carrying LRP-1 gene deletion at an early stage of embryonic development [31]. 

### 4.2. Endocytic Function

 The LRP-1-mediated endocytic internalization of active proteinases linked to the pan-proteinase inhibitor *α*2M represents a general process to eliminate the excess of active proteinases from cellular environment [19, 32]. Here, we review additional LRP-1-mediated endocytosis that occurs independently from *α*2M to regulate extracellular proteinase activities (Table 1).

#### 4.2.1. Serine Proteinases and Inhibitors

 The binding of tPA to cell surface has first been described through PAI-1-dependent [33] and PAI-1-independent [34] receptors. These receptors have been rapidly identified as being LRP-1 [35, 36]. Orth and colleagues [37] confirmed that tPA, under its free form or complexed to PAI-1, binds to LRP-1 to be intracellularly degraded. Also, LRP-1 was shown to mediate the internalization of uPA associated to PAI-1 [31, 38] and PAI-2 [39]. Pro-uPA binds to purified LRP-1 with affinity 15 to 20 fold, weaker than that of the uPA/PAI-1 complex [40]. In contrast, PAI-1 was described to interact with LRP-1 with high affinity when associated with proteinases [41]. These data strongly suggest that the binding of proteinase to PAI-1 could modify PAI-1 conformation, revealing a cryptic high-affinity binding site for LRP-1.

 Besides its ability to link to LRP-1 to be internalized [40], pro-uPA is activated upon binding to uPAR [42]. Interestingly, both uPAR endocytosis and uPA catabolism are dependent on PAI-1 [43]. These important data support the role of LRP-1 in promoting the cell-surface PA activity by facilitating the clearance of uPA/PAI-1-occupied uPAR and the regeneration of unoccupied uPAR at the cell surface [44, 45]. Such a process requires a direct binding between uPAR and LRP-1 [46]. This cycle of binding uPA/PAI-1 to uPAR followed by association with LRP-1, internalization, and intracellular dissociation and recycling of unoccupied uPAR and free LRP-1 to the cell surface can explain, at least in part, the promigratory effect of PAI-1 observed in invasive cells [47]. 

#### 4.2.2. Matrix Metalloproteases and Inhibitors

 In addition to its effect on uPA and tPA activities, LRP-1 also regulates extracellular levels of MMP-2, -9, and -13 [48]. As for uPA and tPA, the endocytosis of MMP-2 and MMP-13 involves preliminary binding to adjacent receptors. Indeed, when bound to thrombospondin-2 (TSP-2), proMMP-2 first associates with an unknown cell-surface heparin-sulfate proteoglycan before interacting with LRP-1 [49]. When complexed with its specific inhibitor TIMP-2, proMMP-2 first binds to an unidentified coreceptor before being internalized by LRP-1 [50]. Likewise, the endocytic clearance of MMP-13 by LRP-1 requires a two-step process, involving a first binding to a 170 kDa co-receptor [51]. The internalization of MMP-9 by LRP-1 requires a more simple mechanism. Thus, proMMP-9/TIMP-1 directly interacts with LRP-1, leading to its endocytic uptake and degradation by lysosomal proteases [52]. The analysis of this interaction reveals that the hemopexin domain of MMP-9 contains a binding site for LRP-1 [53]. 

Although Hahn-Dantona et al. failed to demonstrate a direct interaction between TIMP-1 and LRP-1 in their *in vitro* study [52], our unpublished data reveal that LRP-1 could bind and endocytose TIMP-1 in neurons, in an MMP-independent way. Furthermore, noncomplexed TIMP-2 [50] and TIMP-3 [54, 55] also bind directly to LRP-1 to be internalized. 

#### 4.2.3. Other Matrix Proteinases

 Heparanase-1 is secreted as an inactive heparanase precursor. Once activated, this endoglycosidase degrades heparan sulfate and consequently alters the stability of ECM [56]. The group of Guido David [57] has clearly identified LRP-1 as one of the receptors able to mediate the uptake of secreted heparanase precursor and its intracellular trafficking to the site of activation process. Recently, ADAM with thrombospondin motifs 5 (ADAMTS-5), a major aggrecan-degrading enzyme in cartilage, has been shown to be endocytosed by LRP-1 [58].

 The aspartic proteinase cathepsin-D (cath-D) is capable of degrading ECM in an acidic microenvironment [59]. Recently, Liaudet-Coopman and colleagues [60, 61] identified pro-cath-D as the first ligand of the extracellular domain of LRP-1 *β*-chain. 

### 4.3. Signaling Function

 Additionally, LRP-1 acts in signaling pathways [8, 28, 62]. We recently demonstrated that the abrogation of LRP-1 expression inhibited migration and invasive capacities of thyroid carcinoma cells despite a strong stimulation of pericellular MMP-2 and uPA proteolytic activities [63]. We identified ERK and JNK as the main molecular relays by which LRP-1 regulates focal adhesion disassembly of malignant cells to support invasion [64]. 

 A stimulating study reveals that LRP-1-mediated endocytosis of tPA and tPA/PAI-1 complex is accompanied by a decrease in tPA mRNA transcription [65], suggesting that a secreted protein could regulate its own biosynthesis. Furthermore, the binding of tPA to LRP-1 triggers intracellular signal transduction to induce the expression of another matrix proteinase, MMP-9, both in microvascular endothelial cells [66] and fibroblasts [67]. Probably more surprising, the binding of proteinase inhibitors to LRP-1 also induces MMP-9 expression, as demonstrated for the serpin nexin-1 in a mammary tumor model [68], and activate *α*2M in macrophage-derived cell lines [69]. Recently, the knockdown of LRP-1 expression in human glioblastoma U87 cells revealed that LRP-1 promoted cell migration and invasion by inducing the expression of MMP-2 and MMP-9 [70]. 

 Altogether, these data indicate a close link between MMP-9 and LRP-1: from one of its ligands to a product of LRP-1-induced expression. This suggests important functions for MMP-9 in normal and pathophysiological conditions.

### 4.4. Regulation of LRP-1 Cell-Surface Level and Endocytic Activity by Shedding

 Most membrane proteins, including type I and type II transmembrane proteins, are subjected to a shedding process, that is, the proteolytic cleavage of their extracellular part or ectodomain [71]. LRP-1 also constitutes a membrane target for numerous proteinases. The LRP-1 ectodomain consists in the entire extracellular *α*-chain (515 kDa) noncovalently associated to the extracellular part (55 kDa) of the transmembrane *β*-chain [72]. 

 The product of LRP-1 shedding, the soluble LRP-1 (sLRP-1) *α*-chain, was first detected in human plasma and serum [73]. A metalloproteinase, cleaving LRP-1 at the membrane-proximal region of the *β*-chain, was described in human BeWo choriocarcinoma cells [72]. Since this work was completed, different metalloproteinases have been identified, mainly among the ADAM family. Thus, ADAM-10 and ADAM-17 are associated to LRP-1 shedding in human brain [74]. We recently showed that ADAM-12 exhibited sheddase activity towards LRP-1 in human HT1080 fibrosarcoma cells [75]. Additionally, we reported that MT-MMP, first described to degrade LRP-1 in small fragments [76], was able to generate sLRP-1 in medium conditioned by HT1080 cells in culture [75]. Besides these metalloproteinases, tPA and BACE-1 were described to mediate shedding of LRP-1 [77, 78]. It has been reported that, during cerebral ischemia, tPA induces the shedding of LRP-1 from perivascular astrocytes followed by the development of cerebral edema [79]. These authors demonstrated that the interaction between tPA and LRP-1 in perivascular astrocytes induced Akt phosphorylation, leading to an increase of permeability in the blood-brain barrier. 

 Soluble LRP-1, which is composed of the entire extracellular *α*-chain and noncovalently associated extracellular part of the transmembrane *β*-chain [72], retains ligand-binding capacity and acts as a decoy receptor [80]. Thus, Quinn and colleagues first reported that the addition of sLRP-1 to cultured rat hepatocytes resulted in an inhibition of tPA clearance [73]. Immunoprecipitation assays confirmed that tPA interacted with LRP-1 [72]. Also, LRP-1 shedding from human lung fibroblasts impairs endocytosis of MMP-2 and -9 [81]. We similarly reported that the inhibition of LRP-1 shedding increased MMP-2 and -9 activities, in cultures of human endometrial explants [82] and fibrosarcoma cells [75]. Finally, we recently demonstrated that TIMP-3 bound to sLRP-1, which is resistant to endocytosis, retained its inhibitory activity against metalloproteinases [55].

## 5. Conclusion

Understanding the precise role of LRP-1 in the regulation of ECM breakdown remains an exciting challenge, as it appears to be a multifunctional “Swiss knife.” Thus, besides the endocytosis of proteinases, LRP-1 mediates the clearance of their own inhibitors [8]. Moreover, LRP-1 acts as a membrane receptor that transduces intracellular signals to induce the MMP-9 expression [66–69]. Finally, the proteolytic cleavage of LRP-1 at the cell surface solubilizes the LRP-1 ectodomain, which conserves ligand-binding capacities. Such a property could allow matrix proteinases—but also inhibitors—to increase their extracellular half-life time by escaping from endocytic clearance mediated by membrane-LRP 1. 

 Despite a strong stimulation of pericellular MMP-2 and uPA proteolytic activities, carcinoma cell invasion decreased by LRP-1 silencing [63]. This result clearly indicates that, depending on parameters yet to be elucidated, the signaling function of LRP-1 can counteract or override its endocytic function.

 Another paradox is represented by a proteinase, tPA, for instance, which can either be endocytosed by LRP-1 or solubilize the ectodomain of LRP-1. At the molecular level, interactions between tPA and LRP-1 will be different according to the event: binding to domains 2 and 4 of the LRP-1 *α*-chain for endocytic pathway or cleaving at a single site of both *α*- and *β*-chains at the vicinity of the cell surface for shedding LRP-1 ectodomain. Which is determinant the enzyme or the cell?

 On the whole, these data strongly suggest that LRP-1 does not act alone but with membrane partners, which vary according to numerous parameters including cell origin, ECM composition, pathological conditions, and so forth. In this way, we recently demonstrated [83] that LRP-1 forms complex with the hyaluronan receptor CD44, which may bind proMMP-9 [24]. The identification of these partners could represent a key to the understanding of the LRP-1 roles in ECM remodeling. 

## Figures and Tables

**Table 1:** Main matrix proteinases and specific inhibitors known to bind to LRP-1.

Serine proteinases, serpins, and serine proteinase/serpin complexes		
tPA	PAI-1	tPA, uPA/PAI-1
(pro)uPA		uPA/PAI-2

MMPs, TIMPs, and MMP/TIMP complexes		
(pro)MMP-2/TSP-1, -2	TIMP-1	(pro)MMP-2/TIMP-2 (pro)MMP-9/TIMP-1
(pro)MMP-9	TIMP-2	
(pro)MMP-13	TIMP-3	

Other matrix proteinases		
Heparanase precursor		
Procathepsin-D		
ADAMTS-5		
